# Selection of Immiscible Polymer Blends Filled with Carbon Nanotubes for Heating Applications

**DOI:** 10.3390/polym11111827

**Published:** 2019-11-06

**Authors:** Louis Marischal, Aurélie Cayla, Guillaume Lemort, Christine Campagne, Éric Devaux

**Affiliations:** ENSAIT, GEMTEX–Laboratoire de Génie et Matériaux Textiles, F-59000 Lille, France; aurelie.cayla@ensait.fr (A.C.); guillaume.lemort@ensait.fr (G.L.); christine.campagne@ensait.fr (C.C.); eric.devaux@ensait.fr (É.D.)

**Keywords:** heating textile, conductive polymer composite (CPC), immiscible polymer blends, co-continuity, localization of fillers, Joule effect

## Abstract

In many application fields, such as medicine or sports, heating textiles use electrically conductive multifilaments. This multifilament can be developed from conductive polymer composites (CPC), which are blends of an insulating polymer filled with electrically conductive particles. However, this multifilament must have filler content above the percolation threshold, which leads to an increase of the viscosity and problems during the melt spinning process. Immiscible blends between two polymers (one being a CPC) can be used to allow the reduction of the global filler content if each polymer is co-continuous with a selective localization of the fillers in only one polymer. In this study, three immiscible blends were developed between polypropylene, polyethylene terephthalate, or polyamide 6 and a filled polycaprolactone with carbon nanotubes. The morphology of each blend at different ratios was studied using models of co-continuity and prediction of fillers localization according to viscosity, interfacial energy, elastic modulus, and loss factor of each polymer. This theoretical approach was compared to experimental values to find out differences between methods. The electrical properties (electrical conductivity and Joule effect) were also studied. The co-continuity, the selective localization in the polycaprolactone, and the Joule effect were only exhibited by the polypropylene/filled polycaprolactone 50/50 wt.%.

## 1. Introduction

In the field of smart textiles, the market for heating textiles is growing day by day. Most of these products use metallic yarns [1,2] to ensure their heating properties. However, these metallic yarns can modify the initial textile properties, such as the washability and the hand feel.

One of the solutions consists of using conductive multifilaments processed by melt spinning a conductive polymer composite (CPC), which is a blend composed of an insulating polymer containing electrically conductive fillers. The heating property is provided by the Joule effect due to the electrical conductivity of the textile material [3].

In the literature, several kinds of fillers have been used in order to develop a CPC. The first kind is metallic fillers. As an example, Rivière et al. [4] studied a nanocomposite composed of silver nanowires in a polyetheretherketone matrix. They obtained an electrical conductivity close to 1.45 10^5^ S/m with a filler content of 0.45 vol.%. Another category of fillers is intrinsically conducting polymer (ICP) fillers, which can be used for heating textiles. Zhang et al. [3] showed that using a vapor coating of poly(3,4-ethylenedioxythiophene) (PEDOT) allowed a temperature of 28 °C to be reached with 4.5 V applied on their fabric. The last kind of filler is carbonaceous fillers. Three varieties of carbon fillers are commonly available: graphene, carbon nanotubes, and carbon black. Each of these carbon fillers has different physical and functional properties, such as their shape, specific area, electrical conductivity, and mechanical properties. These three carbon fillers have been studied by many authors concerning the influence of the content of the fillers on the electrical conductivity [5,6,7]. Different variations of carbon black were presented by Kozlowski et al. [8], who showed a difference in electrical and mechanical properties according to the specific area of the carbon black used. Moreover, Xu et al. [9] showed the influence of the aspect ratio and the filler content on the electrical conductivity. Bauhofer et al. [10] showed that it was necessary to have a filler content higher than the electrical percolation threshold. This percolation threshold is the minimum filler content needed in order to create a continuous electrical pathway. After this percolation threshold, the CPC reaches a plateau of high electrical conductivity. In their review, they compiled the electrical percolation threshold for several carbon fillers combined with different polymers, which is lower than 0.1 wt.% in many cases and 0.16 wt.% for polystyrene filled with carbon nanotubes. Miles et al. [11] also showed the importance of polymer viscosity on the dispersion of fillers. Indeed, as Mamunya et al. [12] showed in their study, the fillers dispersion allowed a modification of the percolation threshold. It is necessary to optimize the processing of the CPC due to the many process parameters that can change the electrical conductivity, such as the temperature profile, the rotation speed, and the screw profile [13,14]. However, the filler content can introduce problems during the melt spinning process of the CPC [15,16] due to the increase in the viscosity. Zhang et al. [17] showed the influence of the filler content on the rheological behavior on a poly(ethylene oxide)/poly(methyl methacrylate) blend at ratio 60/40 wt. Straat et al. [18] demonstrated that the viscosity was a key factor in the melt spinning process. In fact, for the melt spinning process to have a viscosity neither too low nor too high, it is necessary to find a compromise between viscosity and electrical conductivity. It is then essential to reduce the percentage of fillers while keeping the electrical conductivity of the final functional polymer. The use of an immiscible blend of polymers (one CPC blended with an insulating polymer) can reduce the global filler content if two conditions are satisfied [19]. A co-continuity of each polymer and the localization of the fillers in only one polymer are both needed in order to enable the electrical conductivity of the CPC with a reduction of the filler content. However, to fulfill these two conditions, it is necessary to control each parameter of the process and choose the correct chemical nature of the polymer blends. Many authors have studied the co-continuity and the selective localization of the fillers. Sumita et al. [20] showed that, in a biphasic blend, there was a double percolation threshold if there was a selective localization of the fillers in two immiscible polymer blends: high density polyethylene/poly(methyl methacrylate) and polypropylene/poly(methyl methacrylate). An immiscible polymer blend can have several morphologies—from dispersed phase to continuous. The percolation threshold is then the phase inversion of one polymer when it changes from the dispersed phase to continuous [21,22]. In their study, the co-continuity was targeted in order to cumulate the properties of each polymer. As the blend reached co-continuity and there was a selective localization of the fillers in only one polymer, a second percolation threshold could be observed in the filled polymer, which was the electrical conductivity percolation threshold, as explained previously. Several models have been created in order to determine the co-continuity of immiscible polymer blends [11,23,24,25,26,27]. In these different models, the co-continuity depends on the properties and the processability of the polymers, such as the rheological properties and the shear stress applied during the process. Thus, it is necessary that, during the process, each parameter—such as the temperature or the pressure [28,29,30,31]—is perfectly controlled to successfully reduce the concentration of the fillers. The localization of the fillers was also studied, for example, by Sumita et al. [32]. They used the wettability coefficient in order to predict the localization of the fillers in an immiscible polymer blend according to the interfacial energy between the components.

In this study, three biphasic blends at different compositions containing nanofillers were studied. The CPC used was polycaprolactone (PCL) filled with multiwalled carbon nanotubes (MWCNT). The second polymer of the biphasic blend was polypropylene (PP), polyamide 6 (PA6), or polyethylene terephthalate (PET). A theoretical approach was made on the co-continuity and the localization of filler for each blend using different models. Then, the morphology of the immiscible polymer blends was tested experimentally by selective phase extraction and rheological measurements. Scanning electron microscopy was also used in order to confirm the morphological observations. The electrical conductivity and the Joule effect of the blends were also studied.

## 2. Materials and Methods

### 2.1. Materials

The first thermoplastic polymer used was polycaprolactone CAPA 6400 supplied by Perstorp (Malmö, Sweden). The melting temperature of this PCL is 60 °C. The second thermoplastic polymer was:

- Polypropylene PPH 9069 supplied by Total (Brussels, Belgium), which has a melting point of 165 °C and a Δ*T* of −0.058 mN/m/K;
- Polyamide 6 Technyl C206 produced by Solvay (Brussels, Belgium), which has a melting point of 222 °C and a Δ*T* of −0.065 mN/m/K;
- Polyethylene terephthalate supplied by Invista (Wichita, KS, USA), which has a melting point of 250 °C and a Δ*T* of −0.065 mN/m/K.

The fillers were multiwalled carbon nanotubes NC 7000 supplied by Nanocyl (Sambreville, Belgium). These MWCNTs have an average length of approximately 1.5 μm, a diameter of 10 nm, and a specific area of 250 m^2^/g.

### 2.2. Compounds Preparations

In order to process each blend, a co-rotating intermeshing twin-screw extruder from Thermo-Haake PTW 16/25p (*Length*/*Diameter* = 25) was used. The rotating speed of this extruder was 100 RPM, and the shear stress during the process was estimated to be close to 20 s^−1^. Before each experiment, PCL was dried at 40 °C and the other polymers at 80 °C for 12 h. Two successive extrusions were applied in order to obtain the functional materials. The first extrusion allowed the incorporation and the dispersion of the MWCNT in the PCL (PCL_MWCNT_). The second step was the extrusion of the filled biphasic blends at different percentages (from 10% to 60% of filled PCL) for each blend: PP/PCL_MWCNT_, PA6/PCL_MWCNT_, and PET/PCL_MWCNT_. The size of the samples were 1.88 mm ± 0.08 mm for the PA6 blend, 1.54 mm ± 0.06 mm for the PP blend, and 1.67 mm ± 0.12 mm for the PET blend

The only differences between each blend during the process were the temperature profiles, which were specific to each preparation. The Table 1 shows the profile temperature of each extrusion.

### 2.3. Methods

#### 2.3.1. Model of Co-Continuity

Three models of co-continuity were used in this study, each of which depends on specific parameters:

• The model of Miles and Zurek [11]:

The model of Miles and Zurek allows one to predict the co-continuity of a blend. This model indicates that the co-continuity is reached when the ratio of volume percentage of polymer 1 and polymer 2 is equal to the ratio of the viscosity in the blending conditions of each polymer. Equation (1) describes the model:

(1) $$\frac{\Phi_{1}}{\Phi_{2}}=\frac{\eta_{1}(\gamma )}{\eta_{2}(\gamma )}$$

where *Φ*_1_ is the volume percentage of polymer 1 in the biphasic blend (%), *Φ*_2_ is the volume percentage of polymer 2 in the biphasic blend (%), *η*_1_ is the viscosity of polymer 1 at shear stress (*γ*) during the process (Pa.s), and *η*_2_ is the viscosity of polymer 2 at shear stress (*γ*) during the process (Pa.s).

• The model of Metelkin and Blekht [27]

This second model predicts the volume percentage of polymer 2 needed in the biphasic blend to reach the co-continuity. This model depends on the viscosity in the blending conditions for each polymer. Equation (2) shows the equation of this model.

(2) $$\Phi_{2}={\left[1+\frac{\eta_{1}}{\eta_{2}}\times \left(1+2.25\times \ln\left(\frac{\eta_{1}}{\eta_{2}}\right)+1.81\times {\left(\ln\left(\frac{\eta_{1}}{\eta_{2}}\right)\right)}^{2}\right)\right]}^{-1}$$

where *Φ*_2_ is the volume percentage of polymer 2 in the biphasic blend (%), *η*_1_ is the viscosity of polymer 1 at shear stress (*γ*) during the process (Pa.s), and *η*_2_ is the viscosity of polymer 2 at shear stress (*γ*) during the process (Pa.s).

• The model of Bourry and Favis [25]

Bourry and Favis developed two equations allowing prediction of the blend co-continuity. Indeed, when the ratio of polymers volume percent is equal to the ratio of polymers elastic modulus (Equation (3)) or equal to the ratio of polymers loss factor (Equation (4)), then the blend is co-continuous:

(3) $$\frac{\Phi_{1}}{\Phi_{2}}=\frac{{G’}_{1}}{{G’}_{2}}$$

(4) $$\frac{\Phi_{1}}{\Phi_{2}}=\frac{\tan\delta_{1}}{\tan\delta_{2}}$$

where *Φ*_1_ is the volume percentage of polymer 1 in the biphasic blend (%), *Φ*_2_ is the volume percentage of polymer 2 in the biphasic blend (%), *G’*_1_ is the elastic modulus (Pa) and tan *δ*_1_ is the loss factor of the polymer 1, and *G’*_2_ is the elastic modulus (Pa) and tan *δ*_2_ is the loss factor of the polymer 2.

#### 2.3.2. Rheological Measurements

A rotational rheometer, AR2000 (TA Instruments, New Castle, DE, USA), with parallel-plate geometry was used to carry out the rheological measurements made in the linear regime. The apparatus performed a shear of 10% in the frequency range from 0.01 to 100 Hz at a constant temperature specific at each blend: 200 °C for PA6, 235 °C for PP, and 265 °C for PET. This experiment allowed for measurement of the viscosity according to the shear stress (γ), the elastic modulus, and the loss factor.

#### 2.3.3. Selective Extraction Experiments

In order to determine the co-continuity of the immiscible polymer blends, a selective extraction was used. Acetic acid allowed for the extraction of the PCL in immiscible polymer blends of PP/PCL_MWCNT_, PA6/PCL_MWCNT_, and PET/PCL_MWCNT_. Before this process, each sample was maintained for 24 h in a room where the temperature and the relative humidity (HR) were controlled (T: 20 °C and H: 65%). They were then immersed in acetic acid at room temperature for 4 h and dried at 50 °C in order to remove the residual acetic acid. Finally, they were returned to the controlled room for 24 h and weighed. This process was repeated in order to find a constant value of sample weight. The PCL accessibility degree (%) was calculated by Equation (5):

(5) $$PCL\;\mathrm{accessibility}\;\mathrm{degree}\;=\frac{W_{i}-W_{f}}{W_{i\;PCL}}\times 100$$

where *W_i_* is the initial weight of the sample (g), *W_f_* is the weight of the sample after PCL extraction (g), and *W_i PCL_* is the initial weight of PCL in the sample before extraction (g).

The *W_i PCL_* was a theoretical value calculated from the initial weight of the sample (*W_i_*) and the percentage of PCL in this sample.

#### 2.3.4. Contact Angle Measurements

The contact angle was measured with a GBX Digidrop (Dublin, Ireland). The contact angle is the angle between the surface of a polymer film with a thickness of 1 mm and a given liquid. For each sample, three different liquids were used in order to measure the interfacial energy: water and α-bromonaphthalene. The liquids’ purities were checked by a GBX tensiometer (Dublin, Ireland). Ten drops were tested with 4.0 μL of wetting liquids and the angles were measured after 20 s at room temperature. Table 2 shows the values of the surface tensions for these two liquids [30].

#### 2.3.5. Interfacial Energy

Fowkes [33] showed that, with the contact angle between a liquid and a solid, it is possible to measure each component of the solid’s surface energy by using Equation (6).

(6) $$\cos\theta =2\frac{\sqrt{{\gamma_{L}}^{D}}}{\gamma_{L}}\times \sqrt{{\gamma_{S}}^{D}}+2\frac{\sqrt{{\gamma_{S}}^{P}{\gamma_{L}}^{P}}}{\gamma_{L}}$$

where *θ* is the contact angle (rad), *γ_L_* is the surface tension of the liquid used (mN/m), *γ_S_* is the surface tension of the surface used (mN/m), *γ_L_^P^* is the polar component of the liquid surface (mN/m), *γ_L_^D^* is the dispersive component of the liquid surface (mN/m), *γ_S_^P^* is the polar component of the solid surface tension (mN/m), and *γ_S_^D^* is the dispersive component of the solid surface tension (mN/m).

Using Equation (6), it is possible to measure the polar component of a solid with a polar liquid and the dispersive component of the solid with a nonpolar liquid. Then, thanks to Equation (7), the surface tension of the solid can be measured.

(7) $$\gamma_{S}={\gamma_{S}}^{P}+{\gamma_{S}}^{D}$$

Cardinaud et al. [34] showed the harmonic equation (Equation (8)) and the geometric equation (Equation (9)), which measure the interfacial energy between components 1 and 2.

(8) $$\gamma_{1-2}=\gamma_{1}+\gamma_{2}-\frac{4{\gamma_{1}}^{D}{\gamma_{2}}^{D}}{{\gamma_{1}}^{D+}{\gamma_{2}}^{D}}-\frac{4{\gamma_{1}}^{P}{\gamma_{2}}^{P}}{{\gamma_{1}}^{P+}{\gamma_{2}}^{P}}$$

(9) $$\gamma_{1-2}=\gamma_{1}+\gamma_{2}-2\sqrt{{\gamma_{1}}^{D}{\gamma_{2}}^{D}}-2\sqrt{{\gamma_{1}}^{P}{\gamma_{2}}^{P}}$$

where *γ*_1-2_ is the interfacial energy between the components 1 and 2 (mN/m), *γ*_1_ is the surface tension of the component 1 (mN/m), *γ*_2_ is the surface tension of the component 2 mN/m), *γ*_1_^*p*^ is the polar component of the component 1 (mN/m), *γ*_1_^*D*^ is the dispersive component of the component 1 (mN/m), *γ*_2_^*p*^ is the polar component of the component 2 (mN/m), and *γ*_2_^*D*^ is the dispersive component of the component 2 (mN/m).

#### 2.3.6. Wettability Coefficient

In the literature, Cardinaud et al. [34] used the wettability coefficient to predict the localization of the fillers in a biphasic blend. The wettability coefficient is described by Equation (10).

(10) $$\omega_{A-B}=\frac{\gamma_{\mathrm{CNT}-B}-\gamma_{\mathrm{CNT}-A}}{\gamma_{A-B}}$$

where *ω*_A−B_ is the wettability coefficient between the components A and B, and *γ*_CNT-B_ is the interfacial energy between the MWCNT and polymer B (Nm/m). The equation shows that:

- If the wettability coefficient is lower than 1, the fillers are localized in polymer B;
- If the result is between −1 and 1, the fillers are at the interface between the two polymers;
- If the wettability coefficient is higher than 1, the fillers are localized in polymer A.

#### 2.3.7. Scanning Electron Microscopy (SEM)

Samples were cut in liquid hydrogen in longitudinal and transverse directions. Then, the samples were carbon metalized with a thickness of 300 Å. Finally, they were observed using SEM images by an SEM Hitachi S4700 operating at 15 kV, 15 mA, and different magnifications, at Commun Microscopie de l’Université de Lille (Lille, France).

#### 2.3.8. Electrical Conductivity Measurement

The electrical conductivity was measured for a length of 1 cm with a Keithley 2461 SourceMeter (Beaverton, OR, USA). This device measures the current intensity while applying a voltage. This voltage ranges from −0.5 V to 15 V with an increment of 0.5 V. Thanks to the voltage and the current intensity, the electrical conductivity can be determined by Equation (11).

(11) $$\sigma =L/\left(R\times S\right)$$

where *σ* is the electrical conductivity (S/m), *R* is the resistance of the sample (Ω), *L* is the distance between the two electrodes (m), and *S* is the cross-sectional area of the sample (m^2^).

#### 2.3.9. Joule Effect Measurement

In order to measure the Joule effect, the Keithley 2461 SourceMeter (Beaverton, OR, USA) and a thermal camera FLIR (Wilsonville, OR, USA) were used. The SourceMeter was connected to two clamps, which maintained the sample at 5 cm above the ground. The distance between the clamps on the sample was 1 cm. A thermal camera, C2 FLIR, connected to the software FLIR (Wilsonville, OR, USA) was placed at a height of 5 cm above the sample. Thanks to the thermal camera and the software, the sample temperature could be measured at any time and everywhere on the sample. The SourceMeter was programmed to deliver a voltage of 20 V for 300 s. Thus, the temperature and the electrical conductivity of the sample could be measured. At least five samples were measured for each blend.

## 3. Results and Discussions

On the one hand, the filler content of the PCL was studied, and on the other hand, the co-continuity, the localization of fillers in the blend, as well as the electrical properties (electrical conductivity and Joule effect) were determined thanks to several experiments. Both theoretical and experimental approaches were used to find out the morphologies. Electrical conductivity and Joule effect measurements were also carried out for each sample.

### 3.1. Study of Filled PCL

**Figure 1:**
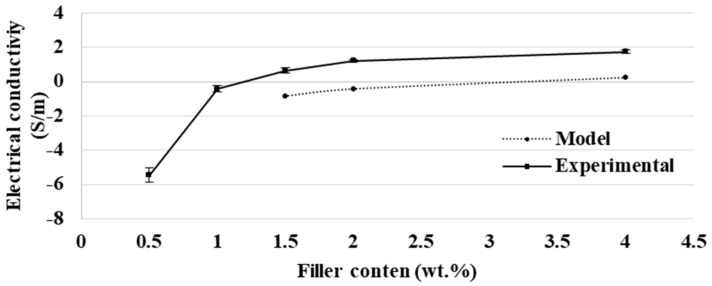
Evolution of the electrical conductivity (log[S/m]) and the model of Kirkpatrick and Zallen according to the filler content of MWCNT in PCL (wt.%).

Filler content of the PCL_MWCNT_ had to be determined before the study of the biphasic blend. The electrical conductivity of filled PCL was measured for a filler content of 0.5/1/1.5/2/4 wt.% in order to find the percolation threshold. In fact, the percolation threshold is the minimum content to have conductive network capabilities of the CPC [6]. Kirkpatrick [35] and Zallen [36] defined a model that allows one to determine the percolation threshold according to the filler content (wt.%). Figure 1 displays the electrical conductivity measurements and the model according to the filler content.

Between 0.5 wt.% and 1.5 wt.% of MWCNT, the electrical conductivity of filled PCL increased sharply, and then the electrical conductivity was stabilizing after 1.5 wt.% of MWCNT. Thus, the percolation threshold, the minimum content filler to have a conductive network, was reached between 0.5 wt.% and 1.5 wt.% of MWCNT. The model of Kirkpatrick [35] and Zallen [36] was also made and confirmed the percolation threshold was between 0.5 wt.% and 1.5 wt.% of MWCNT. However, the filler content influences the viscosity of the blend, and the viscosity is a key factor of the melt spinning process. Thus, it was necessary to have the lowest filler content in order to have a sufficiently low viscosity and an electrical conductivity; the filler content was fixed at 1.5 wt.% in this study.

### 3.2. Study of the Morphology: Co-Continuity

In this study, it was necessary to have co-continuity in each blend in order to reduce the filler content and to have electrical conductivity throughout the product. However, during the process, the immiscible polymer blends may have had several morphologies. In this study, the co-continuity had to be determined for each blend to fulfill the adequate conditions. At first, the co-continuity was determined with different theoretical models: Miles and Zurek [11], Metelkin and Blekht [27], and Bourry and Favis [25]. Each of these models allowed for the determination of the volume percentage of filled PCL needed in order to have co-continuity in each blend: PP/PCL_MWCNT_, PA6/PCL_MWCNT_, and PET/PCL_MWCNT_. As presented in Section 2.3.1, these different models need rheological measurement of the viscosity at shear stress during the process, the elastic modulus, and the loss factor. Rheological values are presented in Table 3.

**Figure 2:**
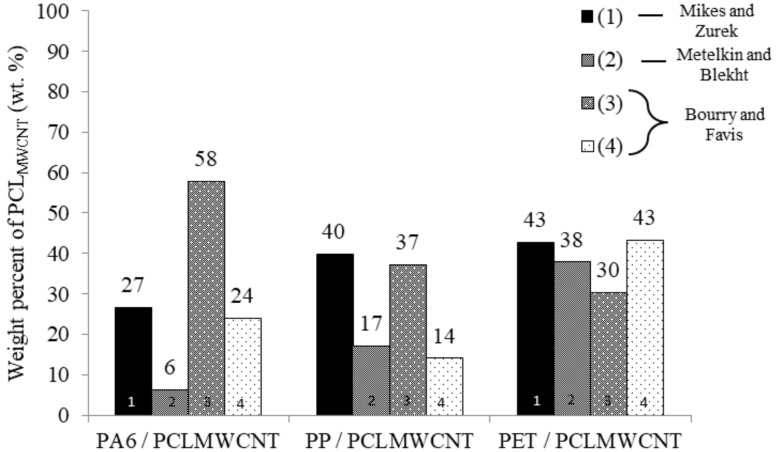
Results of co-continuity of models of Mikes and Zurek, Metelkin and Blekht, and Bourry and Favis for the blend PA6/PCL_MWCNT_, PP/PCL_MWCNT_, and PET/PCL_MWCNT_ according to the weight percent of filled PCL with 1.5 wt.% of MWCNT (wt.%).

Using these values, each model of co-continuity was calculated in order to determine the volume percentage of filled PCL required to have co-continuity. Figure 2 shows the weight percent after a conversion from volume percentage to weight percentage of PCL_MWCNT_ needed to have co-continuity for each model.

**Figure 3:**
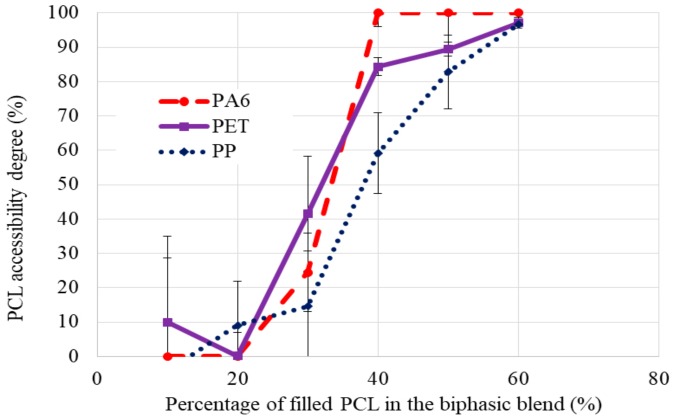
Comparison of percentage of filled PCL with 1.5 wt.% of MWCNT (wt.%) in PA6/PCL_MWCNT_, PP/PCL_MWCNT_, and PET/PCL_MWCNT_ according to the PCL accessibility degree (%).

Thanks to this graph, several conclusions could be made regarding the weight percent of filled PCL required for co-continuity for each blend. For the PA6 blend, there was heterogeneity. Models gave several values to obtain co-continuity from 6 wt.% (model 2) to 58 wt.% (model 3) of PCL_MWCNT_. Thus, no prediction could be made due to these different values. In fact, the purpose of using models was to observe whether the weight percent of PCL_MWCNT_ was the same for each model and so to predict the perfect weight percent of PCL_MWCNT_ to have co-continuity. For the second blend, PP/PCL_MWCNT_, the heterogeneity was lower than it was previously, from 14 wt.% (model 4) to 40 wt.% (model 1) of PCL_MWCNT_. However, no conclusions could be made for the PP/PCL_MWCNT_ blend due to the differences between the models used (1, 2, 3, and 4). Finally, for the last blend, PET/ PCL_MWCNT_, values given by the models were approximately the same, from 30 wt.% to 43 wt.% of PCL_MWCNT_. Therefore, a prediction of co-continuity could be made for the PET blend, and this prediction was between 30 wt.% and 43 wt.% of PCL_MWCNT_. To confirm the theoretical values for the PET blend and find the co-continuity of the two other blends, the co-continuity was evaluated experimentally thanks to a phase selective extraction. Figure 3 shows the results of the PCL accessibility degree (%) for each blend.

Several trends can be observed in Figure 3. First, the PA6 had a PCL accessibility degree close to 0% before 30 wt.% of PCL_MWCNT_. After 30 wt.% of PCL_MWCNT_, the PCL accessibility degree increased to reach 100% at 40 wt.% of PCL_MWCNT_ for the blend PA6. When the PCL accessibility degree attained 100%, the selective phase extraction had extracted all PCL in the biphasic blend. Thus, between 30 wt.% and 40 wt.% of PCL_MWCNT_, a phase inversion could be observed for the PCL from a dispersed to a continuous phase. The co-continuity of the PA6 blend was reached at this moment. The PET blend had a low PCL accessibility degree before 30 wt.% of PCL_MWCNT_. Then, at 30 wt.%, the PCL accessibility degree increased sharply until 100% at 50 wt.% of PCL_MWCNT_. Thus, the phase inversion of the PET blend was reached between 30 wt.% and 50 wt.% of PCL_MWCNT_. For the PP blend before 30 wt.% of PCL_MWCNT_, the PCL accessibility degree was low, but at 40 wt.% of PCL_MWCNT_, the PCL accessibility degree reached 60%. At 50 wt.% of PCL_MWCNT_ for the PP blend, the extraction phase had extracted all of the PCL. Thus, the phase inversion of the PCL_MWCNT_ was between 40 wt.% and 50 wt.% of PCL_MWCNT_, thus the co-continuity of the PP blend was reached in this interval.

**Figure 4:**
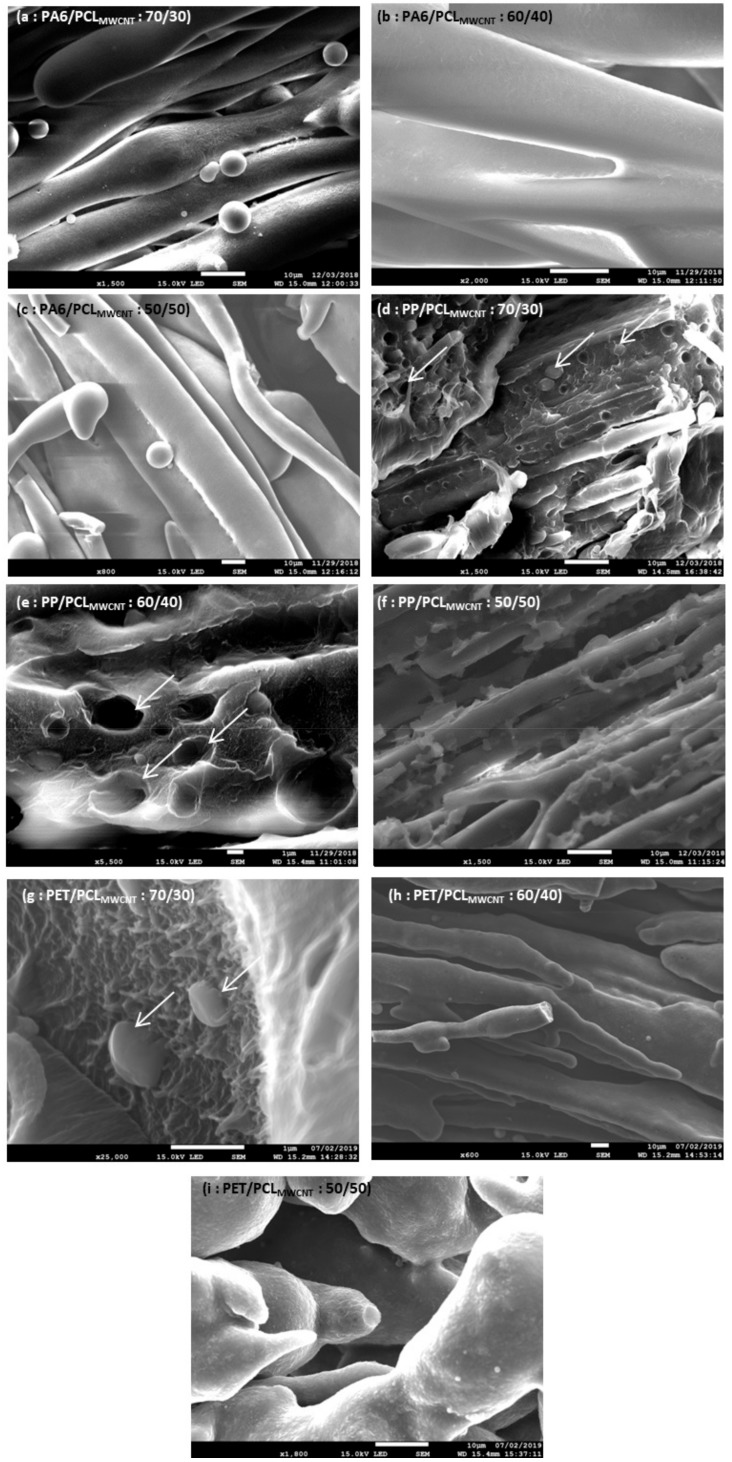
SEM images of PA6/PCL_MWCNT_ (**a**) 70/30, (**b**) 60/40, (**c**) 50/50; PP/PCL_MWCNT_ (**d**) 70/30, (**e**) 60/40, (**f**) 50/50; and PET/PCL_MWCNT_ (**g**) 70/30, (**h**) 60/40, (**i**) 50/50 after phase extraction.

In order to confirm these experimental co-continuity evaluations, SEM images were carried out on samples of each blend after selective phase extraction by acetic acid to extract the PCL in the longitudinal direction. Morphology of each blend at different percentages was observed and is shown in Figure 4.

These observations were carried out in order to confirm the experimental values of each blend. At 30 wt.% of PCL_MWCNT_, there was not a dispersed phase of PCL for the compounds PA6/PCL_MWCNT_ (a), but in the compounds PP/PCL_MWCNT_ (d) and PET/PCL_MWCNT_ (g), dispersed phase of PCL was noted. Thus, the co-continuity was reached for the PA6 blend. These dispersed phases are indicated with an arrow on SEM images. Then, at 40 wt.% of PCL_MWCNT_, the co-continuity could be observed in the PET blend [image (h)]. However, for the PP blend [image (e)], a dispersed phase of PCL was observed. Thus, the PA6 blend reached this co-continuity at or before 30 wt.% of PCL_MWCNT_, contrary to the others blends. For the PET blend, the accessibility degree of PCL predicted that the co-continuity was reached between 30 wt.% and 50 wt.% of PCL_MWCNT_. However SEM images proved that, at 50 wt.% of PCL_MWCNT_, the blend was co-continuous. Thus, the phase inversion of the PET blend was reached between 30 wt.% and 40 wt.% of PCL_MWCNT_ Thus, all models of co-continuity were correctly predicted. The co-continuity of the PP blend was finally reached between 40 wt.% and 50 wt.%. In fact, on the image of the PP blend [image (f)], the co-continuity was noted. Therefore, SEM images allowed us to check the experimental values and the reliability of each model for the three blends. Only for the PET blend did all models give a good approximation of the co-continuity. However, for the two other blends, some models gave good values but were not conclusive due to the different models’ heterogeneity. The conclusion of the utilization of models in our study was similar to that in other studies, such as Castro et al. [37]. It is necessary to remember that each model depends on the selected polymer pair, and each co-continuity value calculated must be verified experimentally. As one of the conditions was the co-continuity, it was necessary to have a ratio of 50/50 between polymers in order to have the co-continuity and to compare each polymer.

### 3.3. Study of the Morphology: Localization of the Fillers

It was necessary to have fillers localized specifically in the PCL in this study. In fact, the goal was to reduce the filler content while keeping the electrical conductivity of the final CPC thanks to a co-continuity of each polymer and the localization of the fillers in only one polymer. To solve this interrogation, the wettability coefficient, which allowed the prediction of the localization of filler, was used [32,34,38]. However, this coefficient had to be used with the interfacial energy at the process temperature in order to predict the final blend: 235 °C for the PA6 blend, 200 °C for the PP blend, and 265 °C for the PET blend. Thus, the interfacial energy at room temperature was calculated with the measurement of the contact angle at room temperature. Next, the interfacial energy at the process temperature was calculated with the value of interfacial energy at room temperature and the value correction factors ΔT (given by Wu [39]), which depend on the materials. Table 4 shows the angle contact between PA6, PP, PET, or PCL_MWCNT_ and water or *α*-bromonaphtalen. All of the values calculated are shown in Table 5.

Using the interfacial energies at the process temperature, the wettability coefficients at the process temperature were calculated and are presented in Table 6.

The wettability coefficients predicted different fillers localization for each blend. This calculation predicted a total MWCNT migration from PCL to PET, a total migration from PCL to PA6, and no MWCNT migration for the PP/ PCL_MWCNT_.

**Figure 5:**
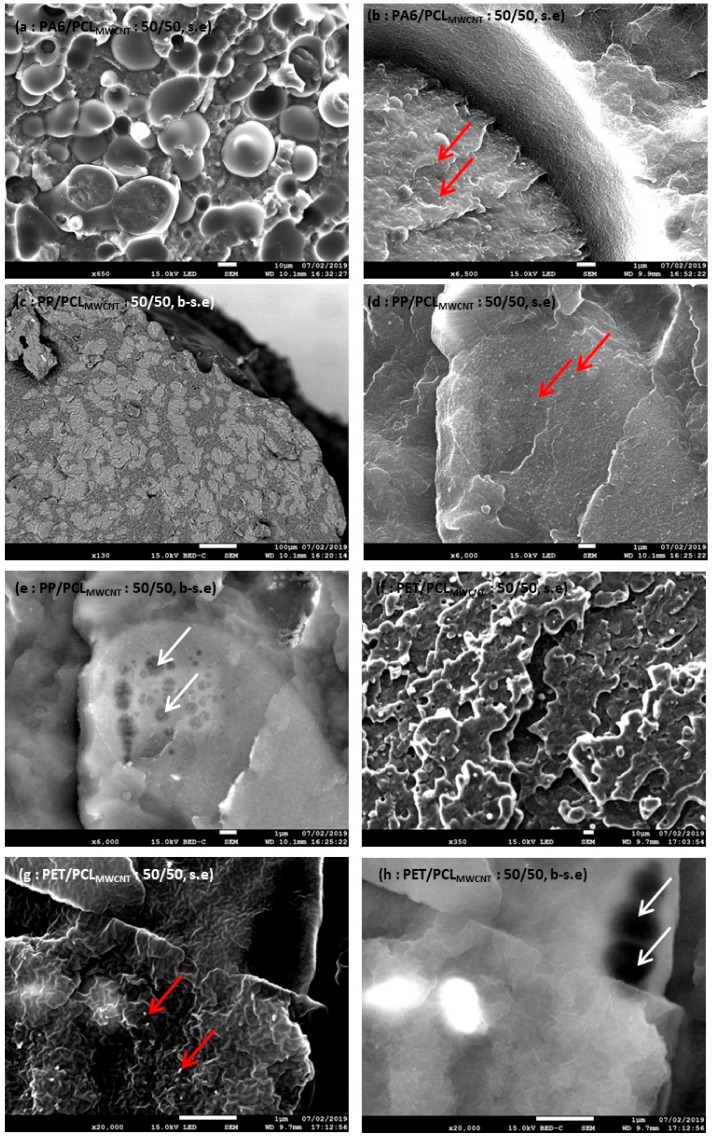
SEM images of PA6/PCL_MWCNT_ 50/50 (**a**,**b**); PP/PCL_MWCNT_ 50/50 (**c**–**e**); and PET/PCL_MWCNT_ 50/50 (**f**–**h**).

Thanks to SEM images (Figure 5) of each blend in the cross-section, it was possible to confirm each prediction.

In images (e), (f), and (h), back-scattered (noted “b-s.e” on SEM images) electrons were observed, as opposed to the other images where secondary electrons (noted “s.e” on SEM images) were observed. These back-scattered electrons allowed us to observe the different polymers according to their density, as in image (f). As the PCL degraded very quickly when the electron beam converged towards it, making a little black hole [as seen in image (h)] with a white arrow, the presence of PCL could be determined in each case. In image (b), it was possible to note that the conductive fillers were only localized in one polymer. Furthermore, this polymer was identified as PA6 due to the fact that it was not degraded quickly. Images (d) and (e) permitted us to note that the fillers (red arrow) were mainly localized in the PCL. Finally, the latest images showed that fillers were localized in the PET. Thus, the SEM images were in agreement with the wettability coefficient predictions. As the second condition was the selective localization of fillers in the PCL, only the PP blend respected this condition as opposed to the two other blends.

### 3.4. Electrical Properties

**Figure 6:**
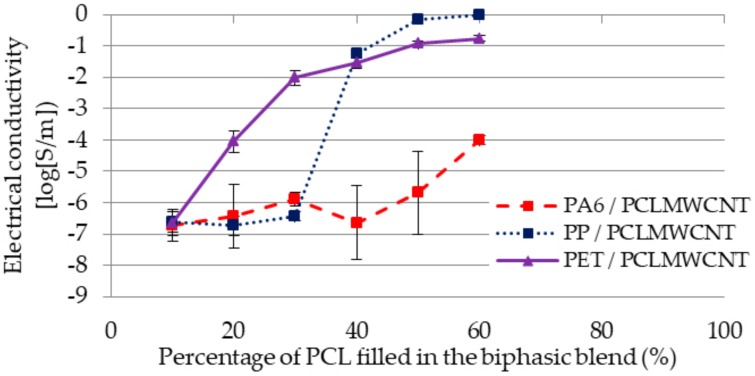
Comparison of the electrical conductivity (log[S/m]) according to the percentage of PCL_MWCNT_ (wt.%) for the blends of PA6/PCL_MWCNT_, PP/PCL_MWCNT_, and PET/PCL_MWCNT._

The electrical conductivity was measured for each blend at different percentages of polymer (Figure 6).

All these results could be explained by the morphology and the selective localization of the fillers. For the PA6 blend, the electrical conductivity was lower than for the two other blends. This electrical conductivity could be explained by the localization of the fillers, which were in the PA6, and by the non-homogeneity of the MWCNT in the polymer. For the PET blend, the electrical conductivity grew directly due to the fact that the fillers in the PCL migrated to the PET. Thus, the more the percentage of filled PCL increased, the more the filler content in the PET increased, involving the creation of the electrical pathways. The PP blend had a low electrical conductivity at the beginning. However, between 30 and 40% of filled PCL, the electrical conductivity grew sharply. This phenomenon was explained by the fact that the co-continuity of PCL was reached at this moment. In fact, as the fillers were located in the PCL, it was necessary for the PCL to be co-continuous in order to create electrical pathways.

**Figure 7:**
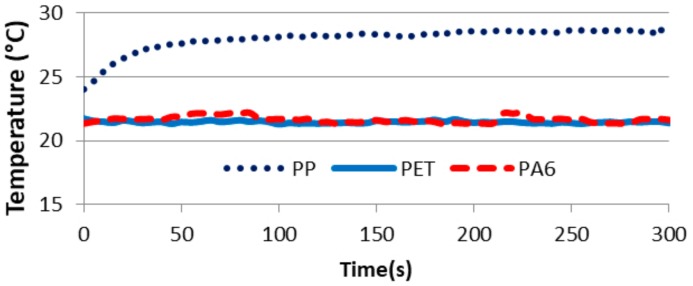
Comparison of the temperature (°C) increase according to the time (s) for the blends of PA6/PCL_MWCNT_, PP/PCL_MWCNT_, and PET/PCL_MWCNT._

Joule effects were measured for these three blends at 50/50 weight percent of polymers. Figure 7 shows the most representative increase of the temperature according to the time for each blend.

Although the electrical conductivity of the PET blend was high, no Joule effect was detected because there was no increase of the temperature. In contrast, the PP blend, which had a higher electrical conductivity, allowed for an increase in the temperature and thus had a Joule effect due to an increase of the temperature of 5 °C. Thus, between the electrical conductivity of the PP blend and the PET blend, there was a Joule effect percolation threshold. In fact, if the electrical conductivity of a sample was lower than the threshold of electrical conductivity allowing the Joule effect, there was no Joule effect, as in the PET blend and the PA6 blend. If the electrical conductivity was higher than this percolation threshold, there was a Joule effect, as in the PP blend. This threshold was defined between the electrical conductivity of the PET bland and the PP blend.

## 4. Conclusions

An alternative method to developing heating textiles is melt spinning with filled PCL. However, during the melt spinning process, high filler content can introduce problems. It is then necessary to decrease the filler content while keeping a maximal electrical conductivity. The solution was the use of immiscible polymer blends with two main conditions: co-continuity of the polymers and selective localization of the fillers in the PCL. Several blends at different percentages were processed by twin screw extrusion: PP/PCL_MWCNT_, PA6/PCL_MWCNT_, and PET/PCL_MWCNT_. The first step for this paper was the study of the co-continuity. Using available models of co-continuity as the models of Mikes and Zurek, Metelkin and Blekht, and Bourry and Favis allowed us to calculate the co-continuity of each blend according to different parameters as the viscosity at blending process, the elastic modulus, and the loss factor. The co-continuity of the PET blend was calculated to be between 30 wt.% and 40 wt.% of PCL_MWCNT_. However, these models could not be predicted for the other blends due to the heterogeneity in the results, from 14 wt.% to 40 wt.% of PCL_MWCNT_ for the PP blend, for example. Experimental values and SEM images confirmed that PET reached this co-continuity between 30 wt.% and 40 wt.% of PCL_MWCNT_ and allowed to us find the co-continuity of the other blends: 30–40 wt.% of filled PCL for the PA6 blend and 40–50 wt.% of filled PCL for the PP blend. The second step was the localization of the fillers thanks the wettability coefficient and SEM images. One the one hand, the wettability coefficient allowed us to make several hypotheses, and on the other hand, these hypotheses were confirmed by SEM observation. In fact, it was observed that each blend had different filler localizations—total migration of the fillers from the PCL to the other polymer for the PA6 and the PET blends and no migration of the fillers from the PCL for the PP blend. After these experiments, to have co-continuity and a localization of filler in the PCL, the blend had to be at a ratio of 50/50 percent of PP/ PCL_MWCNT_. Finally, the electrical conductivity and the Joule effect of each blend with a 50/50 ratio of polymers were measured. Only the PP-based blend allowed a Joule effect, in contrast to the other blends. In conclusion, all of the conditions (co-continuity and selective localization of the fillers in PCL) were respected by the PP blend at 50/50 percent, which also allowed for a Joule effect.

## Figures and Tables

**Table 1:** Temperature profile (°C) of the extrusion of blends: PP/PCL_MWCNT_, PA6/PCL_MWCNT,_ and PET/PCL_MWCNT._

Compound	T_1_ (°C)	T_2_ (°C)	T_3_ (°C)	T_4_ (°C)	T_5_ (°C)
PCL_MWCNT_	55	60	65	70	75
PP/PCL_MWCNT_	110	170	180	190	200
PA6/PCL_MWCNT_	110	170	200	220	235
PET/PCL_MWCNT_	110	150	280	265	265

PCL: polycaprolactone; PP: polypropylene; PA6: polyamide 6; PET: polyethylene terephthalate; MWCNT: multiwalled carbon nanotubes.

**Table 2:** Values of surface tension of liquids: water and *α*-bromonaphtalene.

Liquid	*γ*_L_ (mN/m)	*γ*_L_^D^ (mN/m)	*γ*_L_^P^ (mN/m)
water	72.6	21.6	51
*α*-bromonaphthalene	44.6	44.6	0

**Table 3:** Values of rheological measurement at shear stress of 20 s^−1^ for the PA6, PP, PET, and (PCL_MWCNT: 1.5_)_100._

	Temperature (°C)	Viscosity (Pa·s)	Storage Modulus (Pa)	Loss Modulus (Pa)	Loss Factor
PA6_100_	235	298.59	21,726	55,205	2.54
PP_100_	200	102.81	12,988	14,554	1.12
PET_100_	265	142.64	6264	26,330	4.20
(PCL_MWCNT:1.5_)_100_	200	93.55	24,529	2550	1.04
(PCL_MWCNT:1.5_)_100_	235	104.34	16,436	12,636	0.77
(PCL_MWCNT:1.5_)_100_	265	75.11	13,306	7289	0.55

**Table 4:** Value of angle contact (°) of PA6, PP, PET, and PCL_MWCNT_ with water and α-bromonaphtalen.

Contact Angle (°) between	Water	*α*-bromonaphtalen
PA6	79.3	43.9
PP	112.8	50.5
PET	78.2	39.3
PCL_MWCNT_	82.3	44.3

**Table 5:** Values of interfacial energy (mN/m) of PA6, PP, PET, PCL_MWCNT_, and MWCNT at room temperature and at process temperature.

Materials with Δ*T* (mN/m/K)	Temperature (°C)	*γ*_S_ (mN/m)	*γ*_S^D^_ (mN/m)	*γ*_S^P^_ (mN/m)
PA6: −0.065 ^(1)^	21	38.2	33.0	5.2
PA6: −0.065 ^(1)^	235	24.3	20.9	3.3
PP: −0.058 ^(2)^	21	30.1	29.9	0.2
PP: −0.058 ^(2)^	200	19.6	19.5	0.1
PET: −0.065 ^(2)^	21	40.2	35.1	5.1
PET: −0.065 ^(2)^	265	24.3	21.2	3.1
PCL_MWCNT_: −0.065 ^(1)^	21	37.0	32.8	4.1
PCL_MWCNT_: −0.065 ^(1)^	200	25.3	22.4	2.8
PCL_MWCNT_: −0.065 ^(1)^	235	23.0	20.4	2.6
PCL_MWCNT_: −0.065 ^(1)^	265	21.0	18.7	2.3
MWCNT	21	27.8 ^(1)^	17.6 ^(1)^	10.2 ^(1)^

^(1)^ Values found in the Polymer Handbook, Part IV [40]; ^(2)^ Values found in the study of Koysuren et al. [41].

**Table 6:** Values and predictions of the wettability coefficient (mN/m) on the localization of MWCNT in PA6/PCL_MWCNT_, PP/PCL_MWCNT_, and PET/PCL_MWCNT._

	*ω* PA6/PCL_MWCNT_ at 235 °C	*ω* PP/PCL_MWCNT_ at 200 °C	*ω* PET/PCL_MWCNT_ at 265 °C
Wettability coefficient (mN/m)	11.78	−2.97	4.25
Prediction of fillers localization	PA6	PCL	PET
